# CDC5 Inhibits the Hyperphosphorylation of the Checkpoint Kinase Rad53, Leading to Checkpoint Adaptation

**DOI:** 10.1371/journal.pbio.1000286

**Published:** 2010-01-26

**Authors:** Genevieve M. Vidanes, Frédéric D. Sweeney, Sarah Galicia, Stephanie Cheung, John P. Doyle, Daniel Durocher, David P. Toczyski

**Affiliations:** 1 Department of Biochemistry and Biophysics, University of California San Francisco, San Francisco, California, United States of America; 2 Samuel Lunenfeld Research Institute, Mount Sinai Hospital, Toronto, Ontario, Canada; National Cancer Institute, United States of America

## Abstract

The *Saccharomyces cerevisiae* polo-like kinase Cdc5 promotes adaptation to the DNA damage checkpoint, in addition to its numerous roles in mitotic progression. The process of adaptation occurs when cells are presented with persistent or irreparable DNA damage and escape the cell-cycle arrest imposed by the DNA damage checkpoint. However, the precise mechanism of adaptation remains unknown. We report here that *CDC5* is dose-dependent for adaptation and that its overexpression promotes faster adaptation, indicating that high levels of Cdc5 modulate the ability of the checkpoint to inhibit the downstream cell-cycle machinery. To pinpoint the step in the checkpoint pathway at which Cdc5 acts, we overexpressed *CDC5* from the *GAL1* promoter in damaged cells and examined key steps in checkpoint activation individually. Cdc5 overproduction appeared to have little effect on the early steps leading to Rad53 activation. The checkpoint sensors, Ddc1 (a member of the 9-1-1 complex) and Ddc2 (a member of the Ddc2/Mec1 complex), properly localized to damage sites. Mec1 appeared to be active, since the Rad9 adaptor retained its Mec1 phosphorylation. Moreover, the damage-induced interaction between phosphorylated Rad9 and Rad53 remained intact. In contrast, Rad53 hyperphosphorylation was significantly reduced, consistent with the observation that cell-cycle arrest is lost during adaptation. Thus, we conclude Cdc5 acts to attenuate the DNA damage checkpoint through loss of Rad53 hyperphosphorylation to allow cells to adapt to DNA damage. Polo-like kinase homologs have been shown to inhibit the ability of Claspin to facilitate the activation of downstream checkpoint kinases, suggesting that this function is conserved in vertebrates.

## Abstract 2

The mechanistic role of the yeast kinase CDC5, in allowing cells to adapt to the presence of irreparable DNA damage and continue to divide, is revealed.

## Author Summary

Cellular surveillance mechanisms, termed checkpoints, have evolved to recognize the presence of DNA damage, halt cell division, and promote repair. The purpose of these checkpoints is to prevent the next generation of cells from inheriting a damaged genome. However, after futile attempts at repair over several hours of growth arrest, yeast cells eventually adapt and continue with cell division despite the presence of persistent DNA lesions. This process of adaptation employs CDC5, a kinase that also has essential roles in promoting cell division in the absence of DNA damage. We found that increasing levels of Cdc5 promote adaptation by suppressing the hyperphosphorylation of the checkpoint kinase Rad53, which in turn suppresses the DNA damage checkpoint and relieves cell division arrest. Intriguingly, overexpression of PLK1, the human homolog of CDC5, has been reported in various tumor types and has been linked to poor prognosis. Therefore, understanding the mechanism of adaptation in yeast may provide valuable insight into the role of PLK1 overexpression in tumor progression. Two related papers, published in *PLoS Biology* (van Vugt et al., doi:10.1371/journal.pbio.1000287) and *PLoS Genetics* (Donnianni et al., doi:10.1371/journal.pgen.1000763), similarly investigate the phenomenon of checkpoint adaptation.

## Introduction

Both exogenous pressures and normal cellular processes place stresses on the genome that commonly results in DNA lesions, such as DNA adducts, nicks, and breaks. A robust checkpoint response has evolved to quickly react to the presence of damaged DNA. When triggered, this evolutionarily conserved checkpoint arrests the cell cycle and promotes repair to maintain the integrity of the genome for the next generation of cells. An inability to appropriately repair DNA can lead to mutations, loss of genetic information, or genomic instability [1]–[3].

Checkpoint activation begins with the recruitment of checkpoint sensors to the site of DNA damage. When a double-strand DNA break (DSB) occurs, the DNA ends are resected in a 5′ to 3′ direction by exonucleases, exposing stretches of single-stranded DNA (ssDNA) [4],[5]. The *Saccharomyces cerevisiae* checkpoint sensor complexes, which include the checkpoint clamp and the Mec1 kinase, recognize the exposed ssDNA and accumulate at the break site [6]–[9]. The checkpoint clamp is a ring-shaped heterotrimeric complex that consists of Ddc1, Mec3, and Rad17 (referred to as the 9-1-1 complex) and is reminiscent of the well-studied replication processivity factor PCNA. The 9-1-1 clamp is likely loaded onto DNA at ssDNA-dsDNA junctions that are created by resection, in a manner similar to the proposed mechanism of PCNA loading at sites of replication [10]–[13]. Tel1 accumulates at DSBs and contributes to initial checkpoint activation [9], functioning in parallel with the major yeast sensor kinase Mec1, in contrast to the major role of the mammalian Tel1 homologue, ATM [14],[15]. The Mec1-binding partner, Ddc2, mediates the association with ssDNA by interacting with the ssDNA-binding protein RPA. Similarly, the homologous mammalian kinase, ATR, and its interacting partner, ATRIP, localize to DNA damage via RPA [8].

The co-localization of the checkpoint sensors Mec1 and 9-1-1 leads to the activation of the downstream effector kinases mediated by checkpoint adaptors [16]. Damage created during DNA replication damage utilizes the Mrc1 (Claspin) adaptor to effect checkpoint signaling, whereas damage incurred outside of replication uses the Rad9 adaptor [17]. Upon damage, the Rad9 adaptor is phosphorylated by Mec1, oligomerizes, and serves as a scaffold to promote the activation the effector kinases, Chk1 and Rad53 (Chk2 in mammals) [18]–[23]. Rad9 subsequently mediates Mec1-phosphorylation or “priming” of Rad53, which is required for the auto-phosphorylation of activated Rad53 [22]. In *Schizosaccharomyces pombe*, the phosphorylation of Cds1 (*S.c.* Rad53) by Rad3 (*S.c.* Mec1) is mediated by Mrc1 and promotes a dimerizing interaction through Cds1's fork-head associated (FHA) domain that helps induce Cds1 hyperphosphorylation [24],[25]. This mechanism is likely conserved in *S. cerevisiae* given that the Rad53 FHA domains and Mec1 phosphorylation sites are similarly required for its hyperphosphorylation.

If unable to repair genomic damage, yeast will eventually override the checkpoint and continue with cell division despite the persistence of a break, in a process called adaptation. The *S. cerevisiae* polo-like kinase, Cdc5, was implicated to have a role in adaptation when the *cdc5-ad* allele was identified in a screen for adaptation-defective mutants [26]. This allele has a leucine mutated to a tryptophan at residue 251 and has wild-type activity when assayed on a heterologous substrate [26],[27]. Importantly, the timing of adaptation onset correlated with the loss of Rad53 activity [28], suggesting that adaptation may be a consequence of Cdc5-mediated checkpoint inhibition. Studies in higher eukaryotes provide supporting evidence that polo kinase can inhibit the checkpoint response after DNA damage. The *Xenopus* homolog of Cdc5, Plx1, decreases Chk1 activity by promoting the dissociation of the replication-checkpoint adaptor Claspin from chromatin [29]. Similarly, during recovery after DNA damage, the human Plk1 phosphorylates Claspin to promote its SCF^βTrCP^-dependent degradation, which in turn prevents further Chk1 activation [30]–[32].

In this study, we overexpressed *CDC5* from the GAL1 promoter to probe how Cdc5 interacts with the DNA damage checkpoint to promote adaptation. We found that the checkpoint steps leading to Rad53 activation, including checkpoint sensor localization, Mec1-phosphorylation of Rad9, and formation of the Rad9-Rad53 complex, remained mostly unaffected by Cdc5 overproduction. However, damage-induced hyperphosphorylation of Rad53 was lost and cells reentered the cell-division cycle.

## Results

### *CDC5* Is Dose Dependent for Adaptation

**Figure 1:**
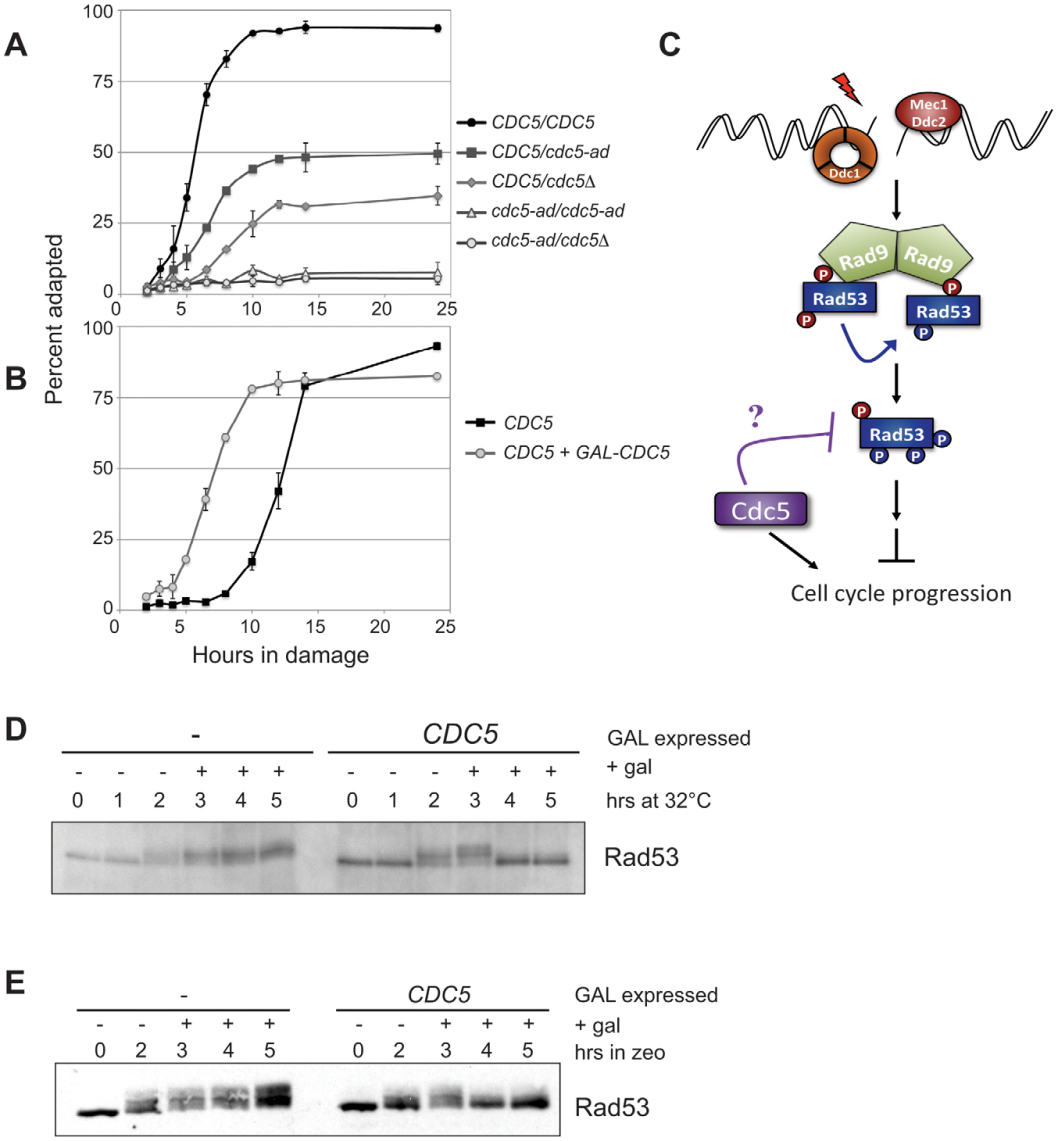
*CDC5* overexpression promotes adaptation by suppressing checkpoint signaling. (A) Adaptation was measured by microcolony assay [33] in diploid strains carrying a combination of *CDC5*, *cdc5-ad*, or *cdc5Δ* alleles, or (B) in haploid strains with or without additional copies of integrated GAL-HA-CDC5. (C) Schematic model of checkpoint signaling. (D) Rad53 was analyzed by Western blots from cells that did or did not overexpress *HA-CDC5* after DNA damage was induced by shifting to the non-permissive temperature of *cdc13-1* strains or (E) by treating cells with 300 µg/ml zeocin. 2% galactose and 10 µg/ml nocodazole were added after 2 h of damage induction.

An allele of *CDC5*, *cdc5-ad*, was originally identified in a screen for adaptation-defective mutants [26]. To determine if the process of adaptation is sensitive to the dosage of *CDC5*, we first analyzed diploid yeast carrying various combinations of *CDC5* alleles: wild-type (WT), *cdc5-ad*, or a deletion. The percentage of cells able to adapt to the DNA damage checkpoint was first measured by creating DNA damage with the *cdc13-1* temperature-sensitive allele. Shifting *cdc13-1* strains to the non-permissive temperature destabilizes telomeres, causing the accumulation of ssDNA and eliciting a checkpoint response. We assayed adaptation by shifting these strains to the non-permissive temperature of 32°C for 2 h, plating cells to pre-warmed plates, and then counting the number of cells able to form microcolonies [33]. As expected and consistent with previous observations, we found that greater than 90% of *CDC5/CDC5* homozygous diploids were able to adapt after 10 h of persistent and irreparable DNA damage (Figure 1A) [26]. Moreover, diploids that express *cdc5-ad* as the only functional *CDC5* allele (*cdc5-ad*/*cdc5-ad* and *cdc5-ad*/*cdc5Δ*) were unable to adapt for the duration of the 25-h time course. However, in heterozygous strains carrying only one copy of WT *CDC5* (*CDC5/cdc5*Δ and *CDC5/cdc5-ad*), the rate of adaptation slows and the number of cells that adapt drops to less than 50%. The slowed rate of adaptation is consistent with the idea that *CDC5* is dose dependent for adaptation. However, the decrease in the total number of cells that adapt likely reflects the decreasing ability of diploids to survive after prolonged cell-cycle arrest. In support of this conclusion, even those arrests that are not associated with viability loss in the short term, such as those induced with temperature-sensitive alleles of the Anaphase Promoting Complex (APC), show loss of viability after about 10 h, around the time that WT cells grown in glucose adapt (D. Toczyski, unpublished data). The observation that a *CDC5/cdc5*Δ strain shows a more significant defect than a *CDC5/cdc5-ad* diploid suggests that the *cdc5-ad* is not functioning as a gain of function mutation. If *cdc5-ad* had 50% activity for adaptation, the *cdc5-ad/cdc5-ad* and *CDC5/cdc5*Δ strains would have an identical capacity to support adaptation. Yet the observation that a *cdc5-ad/cdc5-ad* strain showed a much more pronounced phenotype than a *CDC5/cdc5*Δ suggests that the *cdc5-ad* allele is significantly impaired for *CDC5*'s adaptation activity. The heterozygote contains a mixed population of cells that adapt, as evidenced in Figure 1A, thus complicating a population-based assay like a Western blot. Despite this, examination of Rad53 directly in these diploids (Figure S1) showed that at the time of adaptation (the 6-h time point), strains with fewer copies of WT *CDC5* had higher levels of phosphorylated Rad53.

To further investigate if increased levels of *CDC5* can promote adaptation, we analyzed haploid yeast expressing endogenous *CDC5* or with additional copies of galactose-inducible *CDC5*. Greater than 80% of WT haploid cells adapted by 12 h (Figure 1B). This is slightly later than seen in the previous experiment (Figure 1A), likely because these cells are grown in a poorer carbon source. Thus, as seen previously, *CDC5* overexpression causes re-budding of checkpoint arrested cells [20].

### *CDC5* Suppresses the DNA Damage Checkpoint

Pellicioli et al. [28] provided evidence that the timing of adaptation coincided with a loss of Rad53 hyperphosphorylation. In contrast, cells harboring the adaptation-defective *cdc5-ad* allele remained arrested with an activated checkpoint. This difference does not appear to be mediated by alterations in the levels of Cdc5 or Cdc5-ad during an adaptation time course (Figure S2). These data suggest that an as-yet unidentified step in the DNA damage checkpoint is turned off to allow cells to adapt. However, adaptation is a long and asynchronous process, making studying the molecular mechanism difficult. Moreover, several pathways downregulate the checkpoint to promote adaptation. Therefore, we used the overexpression of *CDC5* as a tool to probe specifically how *CDC5* impinges on the DNA damage checkpoint. We first wanted to determine whether *CDC5* overexpression inhibited the checkpoint pathway itself, or promoted cell-cycle progression non-specifically at a step downstream of the checkpoint. Damage was induced by shifting *cdc13-1* cultures to the non-permissive temperature of 32°C for 2 h, leading to Rad53 phosphorylation. *CDC5* was then induced by adding 2% galactose to strains expressing *CDC5* under the *GAL1* promoter. Nocodazole was added simultaneously with galactose to prevent the adapting cells from reentering the cell cycle. After galactose addition, the hyperphosphorylation of Rad53 dropped significantly in strains harboring the *GAL-CDC5* construct, but not in control strains lacking the construct (Figure 1D). This suppression of the checkpoint was not specific to *cdc13-1*-induced damage. Rad53 hyperphosphorylation also dropped when the DSB-inducing drug zeocin was used (Figure 1E). Together, these data support the notion that *CDC5* promotes checkpoint inactivation.

### Recruitment of Checkpoint Sensors to DSBs Is Unaffected by *CDC5* Overexpression

**Figure 2:**
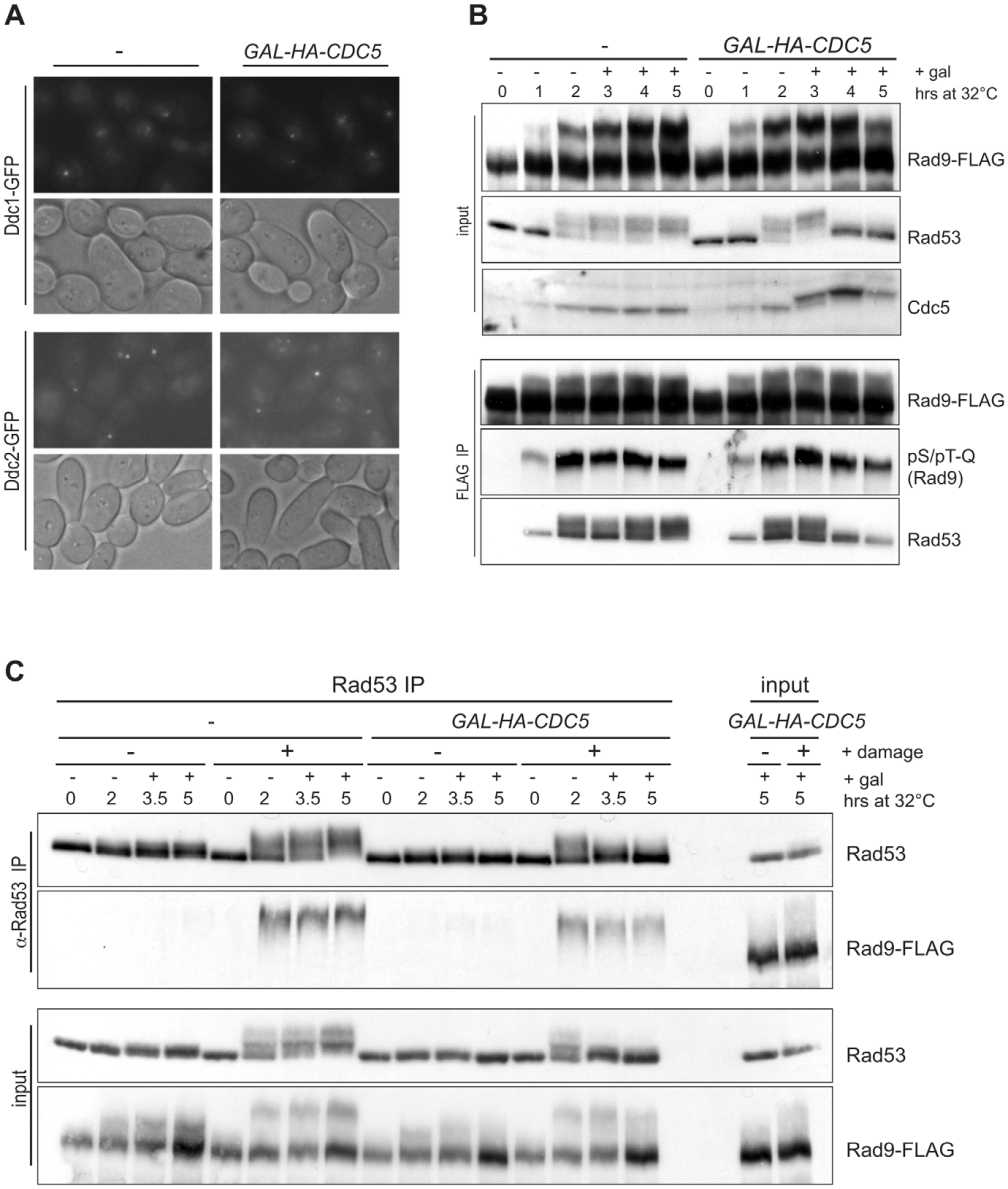
*CDC5* impinges on checkpoint signaling pathway at the step of Rad53 phosphorylation. (A) After 2 h of 300 µg/ml zeocin treatment, 10 µg/ml nocodazole and 2% galactose were added to induce blank or *HA-CDC5* for an additional 2 h. Cells were examined by fluorescence microscopy to visualize Ddc1-GFP or Ddc2-GFP localization. (B) Cells were DNA damaged by shifting *cdc13-1* strains to 32°C for 2 h and then induced to express *HA-CDC5*. Rad9-FLAG was precipitated from lysates with Sigma α-FLAG congugated agarose beads. IP and lysates were analyzed by Western blotting with the indicated antibodies. (C) The reciprocal IP was performed as described in (B), immunoprecipitating Rad53 with the α-Rad53 (DAB001, from the Durocher lab) antibody on Protein A Dynabeads. Strains listed as+/−damage are *cdc13-1* or *CDC13*, respectively.

To determine how *CDC5* suppresses Rad53 phosphorylation, we examined several upstream steps leading to Rad53 activation after *CDC5* overexpression. Recruitment of checkpoint sensors to DSBs is one of the earliest events in checkpoint activation (Figure 1C) and can be visualized by microscopy [6],[9]. Therefore, we monitored the localization of green fluorescent protein (GFP) fused to the checkpoint sensors Ddc1 and Ddc2, a 9-1-1 checkpoint clamp subunit and the Mec1 binding partner, respectively. Cells were treated with zeocin for 2 h before adding galactose to induce *CDC5* for an additional 2 h, as in Figure 1E, and were then examined by fluorescence microscopy. Both Ddc1-GFP and Ddc2-GFP form multiple foci in cells treated for 4 h with zeocin (Figure 2A, left column). Importantly, *CDC5* induction during the second half of the zeocin treatment did not produce an observable change in either Ddc1-GFP or Ddc2-GFP foci formation (Figure 2A, right column) in contrast to its effect on Rad53 phosphorylation at 4 h (Figure 1E). The maintenance of checkpoint sensor localization at break sites, despite *CDC5* overexpression, suggests Cdc5 likely acts downstream of this recruitment step. In previous experiments, Ddc2-GFP foci were found to be lost in a subset of adapted cells at late time points [6]. Our results using *CDC5* overexpression suggest that this is not the result of Cdc5 activity, although it may contribute to adaptation.

### Regulation of the Rad9 Checkpoint Adaptor in Damage Remains Unaffected by *CDC5*

We next investigated if Cdc5 inhibits Rad53 hyperphosphorylation by interfering with the Rad9 checkpoint adaptor. Rad53 activation occurs through the coordination of the adaptor Rad9 and the sensor kinase Mec1 (Figure 1C). Following checkpoint recruitment to DSBs, Rad9 is phosphorylated by Mec1 and, to a lesser extent, Tel1. This phosphorylation promotes Rad9 association with Rad53 [18],[19],[23]. DNA damage-induced Mec1/Tel1 phosphorylation causes a substantial electrophoretic mobility shift in Rad9. This step in checkpoint activation was also largely unchanged by the induction of *CDC5* (Figure 2B, top). To verify that the observed shift in Rad9 was due to phosphorylation by Mec1/Tel1, we probed immunoprecipitated (IP) Rad9 with a phospho-specific antibody that recognizes glutamine directed phospho-serine and phospho-threonine residues, which correspond to Mec1/Tel1 phosphorylation motifs. As expected, the pS/pT-Q antibody only recognized Rad9 after damage induction and with increasing intensity over time (Figure 2B, bottom). As seen in the Rad9-FLAG Western, *CDC5* overexpression resulted in only a subtle change in Rad9's electrophoretic mobility shift. This 2-fold drop in Rad9 hyperphosphorylation was seen at the last (5 h) time point but was not significant at the 4 h time point, despite the fact that Rad53 phosphorylation was already lost by this time. Thus, we conclude that Mec1/Tel1 are able to recognize and phosphorylate Rad9 properly despite *CDC5* induction, suggesting that their kinase activity towards this substrate is not affected.

Cdc5 could disrupt Rad9 function without blocking Mec1/Tel1 phosphorylation of Rad9. First, we determined whether Rad53 remained associated with Rad9 after *CDC5* overexpression. We immunoprecipitated Rad9-FLAG in the presence of DNA damage with or without CDC5 overexpression (Figure 2B, bottom). As previously reported, Rad53 co-immunoprecipitated with Rad9 after induction of DNA damage. Despite the fact that *CDC5* overexpression eliminated Rad53 hyperphosphorylation, Rad53 still associated with Rad9. The reciprocal experiment in which we immunoprecipitated Rad53 showed that only shifted Rad9 binds Rad53. Again, *CDC5* overexpression had a marginal effect on Mec1/Tel1-dependent phosphorylation of Rad9 and had no effect on Rad9's ability to interact with Rad53, despite Rad53's hypophosphorylated state (Figure 2C). Next, we examined the oligomeric state of Rad9. Rad9 has been shown to form a homodimer through its C-terminal BRCT domain [34]. It is possible that *CDC5* could disrupt this higher order structure, thus disabling Rad9's ability to promote Rad53 activation. To determine whether Rad9 multimerization was affected by *CDC5* overexpression, we expressed two differentially tagged alleles of Rad9. Overexpression of *CDC5* did not affect the efficiency with which we were able to co-immunoprecipitate Rad9-myc with Rad9-FLAG (Figure S3). Together, these data suggest that high levels of Cdc5 specifically block the ability of Rad9 to promote Rad53 auto-phosphorylation without affecting the make-up of the Rad9-Rad53 complex.

### Cdc5 kinase Activity Is Required to Suppress Rad53 Hyperphosphorylation

**Figure 3:**
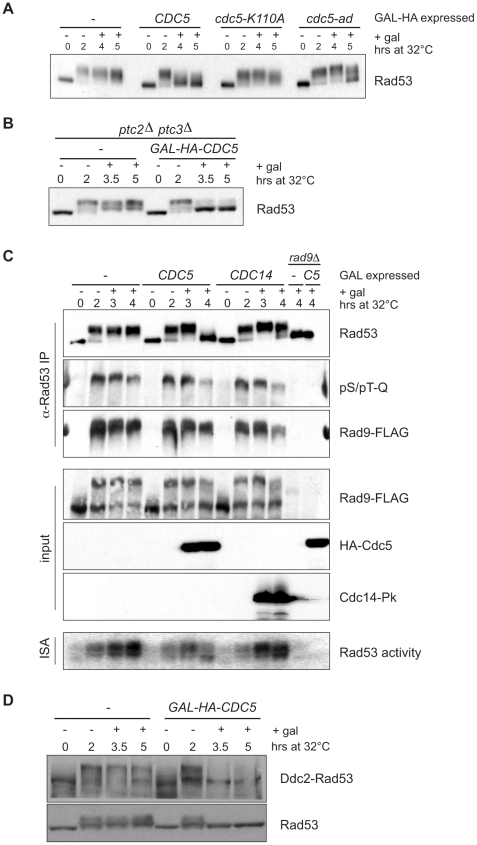
Suppression of Rad53 phosphorylation requires Cdc5 kinase activity but is independent of Ptc2, Ptc3, or Cdc14 phosphatases. Rad53 phosphorylation was examined by Western blot from cells that have been damaged for 2 h before nocodazole and galactose were added to induce (A) *CDC5*, the kinase inactive *cdc5-K110A*, or adaptation defective *cdc5-ad* allele (B), or *CDC5* in cells deleted for the *PTC2* and *PTC3* phosphatases. (C) Rad53 was immunoprecipitated with α-Rad53 from cells treated as above that express either *HA-CDC5* or *CDC14-Pk*. In addition, *rad9Δ* and *rad9Δ GAL-HA-CDC5* strains were examined as controls. Lysates and IP samples were analyzed by Western blotting with the indicated antibodies. (D) The experiment was performed as in parts (A) and (B) excepting that strains were transformed with a construct expressing a Ddc2-Rad53 fusion. Both WT Rad53 and the fusion protein were visualized with anti-Rad53 antibody.

To determine whether the loss of Rad53 hyperphosphorylation requires Cdc5 kinase activity, we compared the effects of overexpressing *CDC*5 and the kinase-defective allele *cdc5-K110A*. Increasing levels of *CDC5* after checkpoint activation resulted in a decrease in Rad53 phosphorylation, as expected (Figure 3A). In contrast, inducing *cdc5-K110A* had no effect (Figure 3A), suggesting Cdc5's kinase activity is necessary for its ability to inactivate checkpoint signaling. Interestingly, induction of the *cdc5-ad* allele produced an intermediate effect, manifested by the later and less robust decrease in Rad53 phosphorylation compared to *CDC5* induction (Figure 3A), consistent with its reduced ability to promote checkpoint adaptation.

### Cdc5 Downregulates the Damage Checkpoint Independently of the Ptc2, Ptc3, and Cdc14 Phosphatases

The PP2C-type phosphatases, Ptc2 and Ptc3, have been implicated to have roles in adaptation and in regulating Rad53 phosphorylation [35],[36]. We generated *ptc2Δ ptc3Δ* strains to test the possibility that *CDC5* acts indirectly on the checkpoint via these phosphatases. If this were indeed the case, we would expect the *ptc2Δ ptc3Δ* stains to be resistant to *CDC5* overexpression. We instead found the damage-induced Rad53 phosphorylation was reduced by *CDC5* induction even in the absence of both Ptc2 and Ptc3 (Figure 3B), implying *CDC5* works independently of these phosphatases. Cdc5 has recently been shown to target *MIH1*, the budding yeast orthologue of the fission yeast *cdc25* phosphatase [37]. As Cdc5 has been shown to inhibit the Swe1 kinase [38],[39], which antagonizes *MIH1*, this phosphorylation may activate Mih1. To test whether Cdc5 is acting through *MIH1*, we deleted *MIH1* and examined the effect of this on Cdc5 mediated Rad53 inactivation (Figure S4A). *MIH1* deletion had no effect on the ability of Cdc5 overexpression to drive Rad53 dephosphorylation. We also examined the effect of *MIH1* overexpression on adaptation itself. To do this, we overexpressed *MIH1* in *CDC5* and *cdc5-ad* disomic strains harboring a non-essential extra copy of chromosome VII with an HO cut site. When HO is induced with galactose, WT strains are able to adapt and grow to form a patch, whereas *cdc5-ad* mutants remain permanently arrested [26],[33]. Overexpression of *MIH1* neither blocked adaptation in WT cells nor rescued the adaptation phenotype in *cdc5-ad* cells (Figure S4B). While Cdc5 seems to act independently of these phosphatases, we cannot rule out the possibility that Cdc5 functions by activating other phosphatases that could mediate the loss of Rad53 phosphorylation.

One of the key roles of the Cdc5 kinase is to advance anaphase by promoting the release of the Cdc14 phosphatase from the nucleolus, which in turn dephosphorylates Cyclin-Dependent Kinase (CDK) substrates. It has been reported that overexpression of *CDC5* results in the premature release of the Cdc14 [40]. Previous work has suggested a role for CDK in checkpoint signaling, in part through its regulation of DNA processing at sites of DNA damage [41]–[44]. To explore the possibility that the loss of Rad53 phosphorylation was a secondary effect of Cdc14 release, we compared the effect of *CDC5* overexpression to *CDC14* overexpression on checkpoint signaling. Similar to that of *CDC5*, overexpression of *CDC14* did not disrupt the damage-dependent interaction of Rad9 and Rad53, although Rad9 displayed a subtle decrease in electrophoretic mobility (Figure 3C).

Lastly, we performed an in situ autophosphorylation assay (ISA). In this assay, total protein is run on a denaturing gel and transferred to a membrane. After renaturation, the membrane is incubated with γ-^32^P. The ISA assay measures autophosphorylation of Rad53 by incorporation of γ-^32^P to membrane-bound renatured Rad53. Surprisingly, *CDC5* overexpression allowed Rad53 to undergo limited autophosphorylation at later time points, although the majority of Rad53 appeared hypophosphorylated (Figure 3C, bottom). In contrast to *CDC5* overexpressing cells, however, Rad53 isolated from *CDC14* overexpressing cells displayed robust autophosphorylation in the ISA assay and a reduction in electrophoretic mobility similar to that of Rad53 isolated from control cells. Together, these results suggest that checkpoint inhibition by Cdc5 cannot be attributed to increased Cdc14 activation.

**Figure 4:**
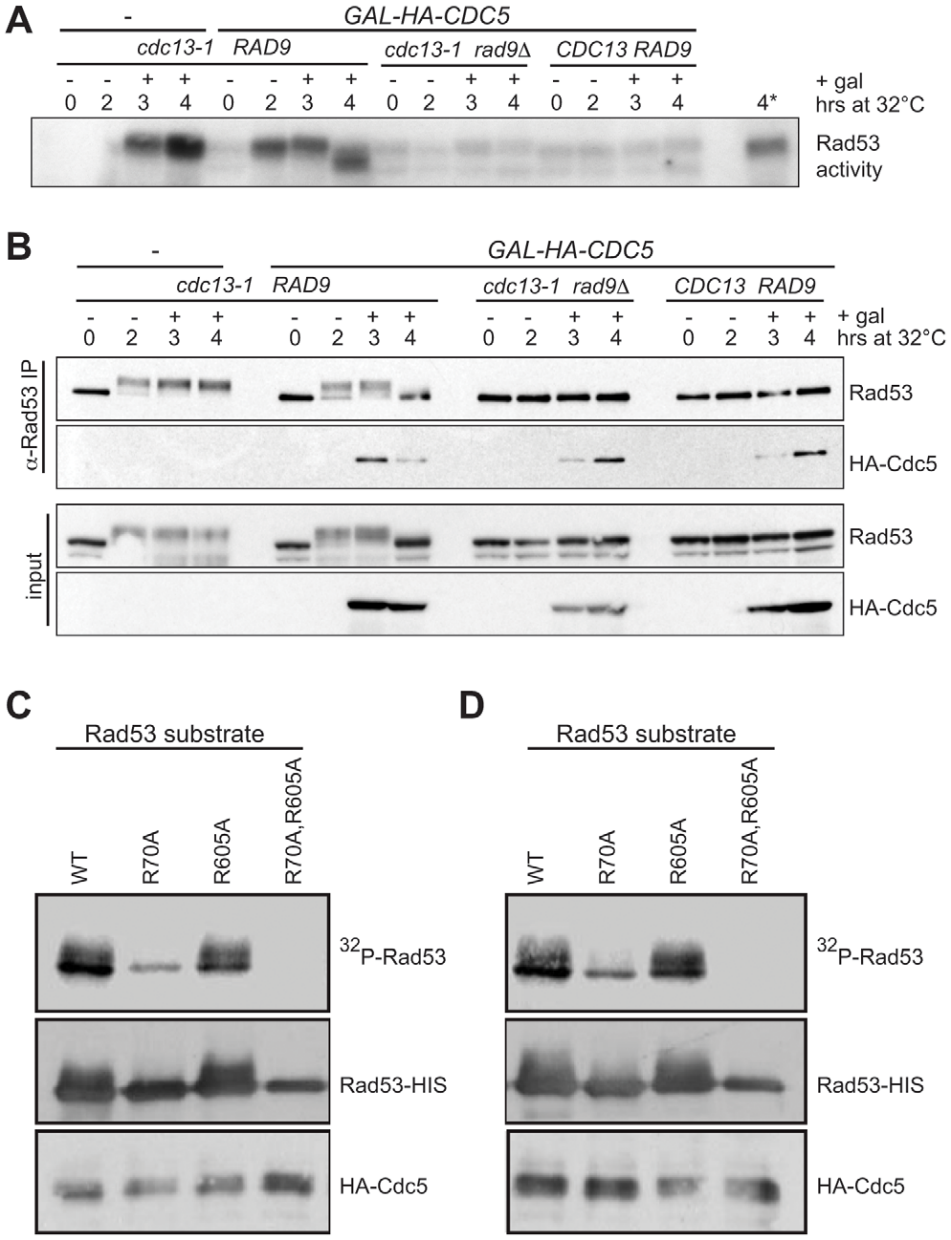
Rad53 interacts with and is phosphorylated by Cdc5. (A) Rad53 was immunoprecipitated from strains shifted to 32°C for 2 h followed by galactose addition to induce the blank or *HA-CDC5*. As added controls, rad9Δ and CDC13 strains were also analyzed. Rad53 activity was measured by in situ autophosphorylation assay from the lysates. Asterisk denotes the lane with half the amount of sample as loaded in lane 4. (B) Lysates and IP samples from the experiment described in (A) were analyzed by Western blotting by the indicated antibodies. (C and D) In vitro kinase assays were performed with purified HA-Cdc5 kinase from (C) undamaged or (D) zeocin treated cells. The substrates were purified recombinant kinase-dead rad53 (D339A, listed as WT) in combination with R70A (FHA1) and/or R605A (FHA2) mutations.

We next attempted to determine whether Rad53, like Rad9, retained its Mec1-dependent priming phosphorylation upon Cdc5 overexpression. Given that Mec1 appeared to retain its activity as judged by Rad9 phosphorylation, we expected that the Mec1-priming phosphorylation on Rad53 was intact. Extensive efforts to examine Rad53 using the phospho-S/T-Q antibodies were unsuccessful, even on purified Rad53 from damage-only control cells. We instead performed an ISA assay and observed that Rad53 appeared to retain its kinase activity, despite the fact that it lost its hyperphosphorylation in vivo (Figures 3C and 4A). This result thus provides an unusual situation in which Rad53 is Rad9 bound and capable of autophosphorylating but rather remains hypophosphorylated in vivo.

While the Rad9-Rad53 interaction persists after Cdc5 induction, it is still possible that Cdc5 overexpression acts not on Rad53 directly, but modifies Rad9 in such a way as to make it unable to promote Rad53 activation. To distinguish between these possibilities, we examined the effect of Cdc5 overexpression on a strain expressing a Ddc2-Rad53 fusion. This fusion was previously shown to bypass the requirement for the adaptors Rad9 and Mrc1 and allow Rad53 to be activated in their absence [45]. We found that the activation of the Ddc2-Rad53 fusion is partially inhibited by Cdc5 (Figure 3D). This suggests that the effect of Cdc5 overexpression on Rad53 activation is not entirely dependent upon Rad9 but may act redundantly upon both Rad9 and Rad53. Importantly, activation of the Ddc2-Rad53 fusion does not require the WT Rad53 [45], so its dephosphorylation cannot be an indirect effect of inactivation of the WT Rad53 allele.

### Cdc5 Binds and Phosphorylates Rad53

Polo-like kinases recognize substrates that have been previously phosphorylated by other kinases, such as CDK and ATM/ATR in higher eukaryotes [29]–[32],[46]. Since Rad53 is phosphorylated by the CDK and Mec1/Tel1 kinases [16],[47],[48], we wondered whether Rad53 could serve as a direct substrate for Cdc5. We first determined if Cdc5 was able to interact with Rad53 in vivo. In fact, the human homolog of Rad53, Chk2, has been reported to directly bind the human Plk1 [49],[50]. HA-Cdc5, as well as the kinase dead HA-cdc5-K110A, was indeed found to immunoprecipitate with Rad53 (Figure 4B and unpublished data) and did not immunoprecipitate in a control *rad53Δ* strain (Figure S5A). To examine whether the interaction of Rad53 and Cdc5 during an active DNA damage checkpoint is mediated by the Rad9 adaptor protein, we also performed the co-immunoprecipitation in a *rad9Δ* strain. HA-Cdc5 was still co-immunoprecipitated with Rad53 in the absence of Rad9 (Figure 4B), suggesting the binding between Cdc5 and Rad53 is not mediated by Rad9. The in vivo interaction between Rad53 and Cdc5 was also found to occur independently of damage (Figure 4B), which is consistent with both the Rad9-independent binding data (Figure 4B) and the human Chk2-Plk1 interaction data [49],[50].

Having discovered that the Cdc5 kinase activity is critical for its function to reduce Rad53 phosphorylation, we performed in vitro kinase assays to determine if Rad53 could be a direct substrate of Cdc5. The HA-Cdc5 kinase was isolated from yeast extracts that were either untreated or damaged with zeocin. To ensure that the in vitro phosphorylation of Rad53 was specific to Cdc5 kinase activity and not a product of Rad53 autophosphorylation, the Rad53 substrates used harbored the D339A kinase inactivating mutation. This Rad53-D339A substrate was otherwise WT (labeled WT) or also carried additional mutations in one or both of the FHA domains. Rad53 contains two FHA domains that are important for checkpoint function [19] and mediate association with phosphorylated proteins, such as Rad9 [51] and potentially Cdc5. The rad53 R70A mutation corresponds to the N-terminal FHA1 domain and the R605A mutation to the C-terminal FHA2 domain. Similar to the in vivo binding data, the in vitro phosphorylation of Rad53 by Cdc5 can occur independently of the DNA damage. HA-Cdc5, isolated either from untreated extracts (Figure 4C) or DNA-damaged extracts (Figure 4D), clearly phosphorylated Rad53 in vitro, as seen by both the incorporation of radio-labelled phosphate or the Cdc5-induced electrophoretic shift of Rad53. As expected, this result required a functional HA-Cdc5 since no Rad53 phosphorylation was observed when the kinase dead mutant, HA-cdc5-K110A, was used as a control (Figure S5B). The rad53-R605A mutant seemed to be phosphorylated to a similar level as the wild-type. Surprisingly, the rad53-R70A mutant alone was less phosphorylated and the R70A R605A double mutant was not at all phosphorylated by Cdc5 (Figure 4C and 4D). These data suggest that the Rad53 FHA1 phosphobinding domain and to a lesser extent the FHA2 domain promote Cdc5's ability to phosphorylate Rad53. Since mutations of either FHA domain compromise (FHA1) or eliminate (FHA2) checkpoint function [19],[52], we were unable to examine the effect of loss of these domains on the ability of cells to adapt to the checkpoint.

## Discussion

Polo-like kinases participate in several processes that collectively promote mitotic progression, including mitotic exit, early anaphase, APC activation, and sister chromatid separation [27],[53]–[55]. The discovery of an adaptation-defective allele of *CDC5* suggested that this kinase also had a role in negatively regulating the DNA damage checkpoint [26], however the mechanistic details remained unknown. Our data suggest that Cdc5 does not inhibit formation of the Rad9-Rad53 complex and yet blocks the ability of the Mec1-primed Rad53 molecules to produce hyperphosphorylated Rad53 in vivo.

Adaptation to DNA damage begins to occur after approximately 6–8 h of cell cycle arrest if cells were unable to repair the damage. Loss of checkpoint signaling has been previously shown to correlate with the onset of adaptation [28]. However, there could be multiple pathways converging on the checkpoint after an extended cell-cycle arrest. One of the advantages of the *CDC5* overexpression approach taken here is that it has allowed us to isolate *CDC5*-specific effects from those of other pathways. For example, Ptc2 and Ptc3 clearly have a role in Rad53 regulation, and deletion of these phosphatases causes an adaptation-defective phenotype [35]. However, we found that checkpoint suppression caused by *CDC5* overexpression occurred in the absence of both these phosphatases (Figure 3B), consistent with the model that at least two pathways work independently to promote adaptation. Ptc2 and Ptc3 may have important roles in recovery from the checkpoint once damage has been repaired. Cdc5 does not appear to have an essential role in the process of recovery, since *cdc5-ad* mutants are able to reenter the cell cycle once damage is repaired [26] and Rad53 dephosphorylation occurs in DNA-damaged *cdc5-ad* strains if *MEC1* activity is removed [28]. Rad53 has recently been proposed to act in a negative-feedback loop, in which Rad53 phosphorylates Rad9 to prevent the BRCT-SCD domain-specific oligomerization of Rad9 that is required to maintain checkpoint signaling [56]. While this negative-feedback loop may also feed into adaptation, our results showing that overproduced Cdc5 prevents in vivo Rad53 autophosphorylation suggest Cdc5 exerts its effect upstream of this loop.

We found that Cdc5 and Rad53 could interact both in vivo and in vitro, which could support the notion that Cdc5 directly inhibits Rad53. Cdc5 kinase activity was required to suppress Rad53 phosphorylation (Figure 3A) and kinase-dead Cdc5 was co-immunoprecipitated with Rad53 (unpublished data), eliminating the mechanism of simple binding inhibition. Interestingly, hypophosphorylated Rad53 from Cdc5 overproducing cells retained its ability to trans-autophosphorylate by ISA (Figure 4B). A population of Rad53 that is capable of undergoing limited autophosphorylation in the ISA assay in the absence of a significant phospho-shift as measured by gel mobility assay has been observed previously in the checkpoint-defective rad53 FHA2-R605A mutant [22],[52]. These data and those presented here suggest that these two assays measure distinct aspects of Rad53 activation that are together required for its in vivo function and that Cdc5 may specifically act to counter one of these functions in vivo. In fission yeast, phosphorylation of Cds1 (the Rad53 homolog) by Rad3 (the Mec1 homolog) is thought to promote Cds1-Cds1 interactions required for autophosphorylation [24]. Similarly, Rad53 autophosphorylation activity requires Mec1/Tel1 phosphorylation [22]. Therefore, if Mec1/Tel1 were completely inhibited, Rad53 would not be active in the ISA assay, which we did not observe. Consistent with Rad53 maintaining its priming phosphorylation, Rad53 remained a tight doublet even after *CDC5* overexpression caused loss of its hyperphosphorylation. This, along with our data demonstrating Rad9 is appropriately phosphorylated by Mec1/Tel1 despite *CDC5* overexpression, would suggest that Mec1/Tel1 are active and can phosphorylate Rad53 enough to prime its activity.

Cdc5 was able to directly phosphorylate Rad53 in vitro. Cdc5 phosphorylation might affect the positioning of Rad53 with respect to either other Rad53 molecules or Rad9 so as to prevent proper Rad53 trans-autophosphorylation. Active and phosphorylated Rad53 must be released from Rad9 [57], suggesting that these Rad9-bound hypophosphorylated Rad53 molecules could act dominantly to prevent further checkpoint activation, as does expression of the kinase-dead allele of *RAD53* [58].

Our demonstration that Cdc5 phosphorylation of recombinant Rad53 depends on both Rad53 FHA domains (Figure 4C and 4D) is particularly intriguing. First, it suggests that this activity is quite specific. Moreover, it argues that Rad53 provides the binding specificity to allow Cdc5 to phosphorylate it, in contrast to the classic model in which polo-like kinases recognize a substrate via their phosphobinding polo-box domains and then subsequently phosphorylate the bound substrate [59]. This mechanism is also different from how the human homologs, Chk2 and Plk1, are reported to interact [50]. However, as both proteins contain phosphobinding motifs, mutual recognition between Cdc5 and Rad53 may be required in vivo. An alternative model of indirect inhibition is one in which Rad53 could bridge an interaction between Cdc5 and Rad9 and promote Cdc5 phosphorylation of Rad9. As a result, Cdc5-mediated phosphorylation could interfere with proper Rad53 autophosphorylation. This model has the benefit of targeting the checkpoint mediator responsible for activating the two parallel effector kinases Rad53 and Chk1, both shown to lose activity as cells adapt [28].

Cdc5 can now be added to the growing list of proteins that interact with the Rad53 FHA1 domain. Rad53 contains two FHA domains, one at each terminus, whereas homologous proteins such as human Chk2 and *S. pombe* Cds1 contain only one N-terminal FHA domain. Although both Rad53 FHA domains contribute to its checkpoint function, the N-terminal FHA1 is more structurally similar to its homologous counterparts. This raises interesting prospects on how Rad53's FHA1 domain facilitates interactions with downstream targets including Dbf4, Asf1, Mdt1, Rad9, and other Rad53 molecules [19],[60]–[63], as well as promote its own inactivation by interacting with Ptc2 [35],[36] and, potentially, Cdc5.

Our results strongly suggest the polo-like kinase, Cdc5, can inhibit checkpoint signaling at the level of Rad53 hyperphosphorylation. Rad53 autoactivation provides an amplification step in which primed Rad53 can activate additional Rad53 molecules in a positive-feedback loop, thereby preventing premature or unnecessary checkpoint activation. The findings that both the in vivo interaction and the in vitro phosphorylation of Rad53 by Cdc5 imply that there is potential for a constitutive interaction, in agreement with human Chk2 and Plk1 data [50]. While the biological significance for a constitutive interaction is not yet clear, it presents the opportunity for each kinase to inhibit the other and generate a switch-like decision to undergo adaptation. Indeed, Plk1 has been reported to be inhibited by the DNA damage checkpoint [64],[65]. This leads us to question, what can tip the balance of this potential inhibitory face-off: the activity of a third kinase such as CDK on either or both Rad53 and Cdc5, or the relative strength of their interaction compared to other substrates?

Adaptation can be considered as a final attempt at survival after yeast have exhausted all other repair options. However, as a consequence of promoting cell division in the presence of DNA damage, adaptation also results in increased genomic stability [66]. Our study of adaptation, particularly our use of *CDC5* overexpression, may provide valuable insights into the mechanisms of tumorigenesis. The human homologue *PLK1* has been reported to be overexpressed in various tumors including non-small-cell lung cancer, melanoma, colorectal cancer, and non-Hodgkin lymphoma. In addition, the levels of *PLK1* in a subset of tumor types may provide prognostic value [67],[68]. Our work implies that, if indeed parallel with adaptation, *PLK1* overexpression could lead to checkpoint suppression, an enhanced rate of mutagenesis due to genomic instability, and ultimately carcinogenesis.

## Materials and Methods

### Yeast Strains and Plasmids

All haploid strains were derived from yDPT1-1 (LS *MatΔ cdc13-1 cyh1 can1 lys5 ade2 ade3::GalHO trp1 his3 ura3 leu2 pep4::LEU2*) and yDPT42-4 (LS *MatΔ cdc13-1 cyh1 can1 lys5 ade2 ade3::GalHO trp1 his3 ura3 leu2 pep4::LEU2 URA3::Gal-HA3-CDC5 2 copies*). Plasmids pDM164, pDM173, and pDM191 were linearized with NcoI to integrate *GAL-HA3-CDC5*, *GAL-HA3-cdc5-K110A*, and *GAL-HA3-cdc5-ad*, respectively. Plasmid c518 was linearized with XcmI to integrate *GAL-Cdc14-Pk*. A PCR-based integration cassette was created to insert a C-terminal 3×Flag epitope tag to *RAD9* using the p3FLAG::HYG. GFP fusions to *DDC1* and *DDC2* were created as described previously [6]. DDC2-RAD53 3× FLAG from the pRS316 DDC2-RAD53 3× FLAG plasmid was cloned into pRS304 and digested with SexAI for integration at the *RAD53* locus [45].

### Fluorescence Microscopy

Cells were treated with 300 µg/ml zeocin (Invitrogen) for a total of 4 h. After the first 2 h of zeocin treatment, 2% galactose and 10 µg/ml nocodazole were added and incubated for another 2 h. Microscopy was performed with the Leica DMRXA microscope using the FITC filter, 100× 1.4NA PlanApo oil-immersion objective, and Hamamatsu C4742-95 CCD camera. Openlab 4.0.3 imaging software (Improvision) was used to capture multiple z-section images. Fluorescence exposure times were kept constant between strains carrying a particular GFP fusion.

### Adaptation

Adaptation was assessed morphologically by counting the number of cells in a microcolony, as previously described [33]. Arrested, large-budded cells were counted as two cells. Additional budding beyond the two-cell stage was considered adapted.

### Rad53 and Rad9 IP

5×10^8^ cells were collected for each IP. The Rad53 IP was carried out with 1 µl/IP of polyclonal DAB001 (gift from D. Durocher) on protein A Dynabeads (Invitrogen), as previously described [22]. For Rad9-FLAG purification, cells were subjected to glass bead lysis at 4°C in lysis buffer (25 mM HEPES-OH, pH 7.5, 250 mM NaCl, 0.2% Triton X-100, 1 mM EDTA, 10% glycerol, and protease inhibitor cocktail). Fifteen µl of Sigma Anti-FLAG M2 agarose beads were added to each sample and allowed to incubate at 4°C for 2 h. The beads were washed four times with lysis buffer. The beads were boiled in SDS-PAGE loading buffer to elute bound proteins.

### Western Blot Analysis

Protein samples were run on 6% or 8% SDS-PAGE gels and transferred onto nitrocellulose membrane (Millipore). α-Cdc5 (YN-19 from Santa Cruz) or α-HA antibodies were used against HA-Cdc5 when listed as Cdc5 or HA-Cdc5 in figures, respectively. Other antibodies used for Western blots include: α-Pk, α-myc, α-FLAG, α-Rad53 (YC-19 from Santa Cruz), and α-pS/pT-Q (Cell Signalling).

### Rad53 and Cdc5 Kinase Assays

The Rad53 purification and ISA were performed as previously described [22],[58]. HA-Cdc5 IP were performed by growing 250 ml cultures of the appropriate strains to OD_600_ = 1 in rich media containing 2% raffinose. Protein expression was induced for 3 h following addition of 2% galactose. Cells were lysed in RIPA buffer (150 mM NaCl, 1% NP-40, 0.5% deoxycholic acid, 0.1% SDS, 50 mM Tris pH 8.0, protease inhibitor cocktail (Roche), and phosphatase inhibitor cocktail (Sigma)). IPs were performed using HA-coupled dynabeads (Invitrogen) for 1 h. The beads were washed three times in RIPA buffer and two times in kinase buffer (25 mM Hepes pH 7.5, 250 mM NaCl, 20 mM MgCl_2_, 20 mM MnCl_2_, 1 mM DTT). In vitro kinase assays were performed as follows. Purified recombinant rad53-D339A was incubated with immunoprecipitated Cdc5 in kinase buffer (25 mM Hepes pH 7.5, 250 mM NaCl, 20 mM MgCl_2_, 20 mM MnCl_2_, 1 mM DTT, 40 mM ATP, and 0.5 ml [γ-^32^P]ATP (Perkin-Elmer)) for 30 min. The reactions were stopped by adding SDS sample buffer and by boiling the sample for 5 min. Half of the reactions were then loaded on an 8% SDS-PAGE gel and transferred to PVDF membrane (Millipore). The membrane was exposed overnight on a phosphor screen (GE Bioscience) and revealed by phosphorimaging (GE Bioscience). All quantifications were performed with ImageQuant 5.0.

## Supporting Information

Figure S1 **Rad53 phosphorylation in diploids.** *cdc13-1* strains were grown overnight at permissive temperature (23°C) in rich media containing 2% dextrose, diluted to an OD_660_ of 0.2, and shifted to 32°C to induce damage. Cells were collected every 2 h. Alpha factor (10 µg/ml) was added to the culture at 4 h with additional boluses at 6 and 8 h to arrest adapting cells in the subsequent G1 phase. Lysates were prepared for Western blot analysis to compare levels of phosphorylated Rad53. yDPT27-2 is *CDC5/CDC5*; yDPT18-9 is *CDC5/cdc5*Δ; yDPT28-3 is *CDC5/cdc5-ad*; yDPT19-19 is *cdc5-ad/cdc5*Δ; yDPT29-1 is *cdc5-ad/cdc5-ad*.
(0.40 MB TIF)

Figure S2 **Levels of Cdc5 are unaffected by adaptation.** (A) Adaptation was measured by microcolony assay in *cdc13-1 CDC5* and *cdc13-1 cdc5-ad* haploid strains. Cells were initially synchronized in G1 with 7.5 µg/ml of alpha-factor at 23°C for 2 h before release into pre-warmed liquid YM-1 at 32°C to induce damage. Cells were plated 2 h after the temperature shift and counted every hour thereafter. (B) Hourly samples were taken from the adaptation time course described in panel A to measure levels of Rad53, Cdc5, and Cdc28, as a loading control, by Western blot. Asynchronous cells are labeled as A; alpha-factor arrested cells are labeled as αf.
(0.47 MB TIF)

Figure S3 **Rad9-Rad9 interaction unaffected by** ***CDC5*** **overexpression.** Rad9-FLAG was immunoprecipitated from strains containing a copy of each RAD9-FLAG and RAD9-18myc that were damaged for 2 h at the non-permissive temperature for *cdc13-1*, then treated with galactose to induce *HA-CDC5*. The 5^m^ and 5^F^ denote the 5 h time point of strains that express only RAD9-18myc or RAD9-FLAG, respectively. Input and IP samples were analyzed by Western blotting with the indicated antibodies.
(0.58 MB TIF)

Figure S4 **Cdc5 does not regulate adaptation through Mih1.** (A) Rad53 phosphorylation was examined after Cdc5 overexpression in wild-type cells, or cells deleted for *MIH1*, as in Figure 2C. (B) All strains are disomic *rad52*Δ mutants carrying a galactose-inducible HO endonuclease and a site for the HO endonuclease on the end of a second copy of chromosome VII (see [26],[33] for complete description). *CDC5* and *cdc5-ad* strains were transformed with CEN-based plasmids lacking an insert (“empty vector”) or with a PGK or Gal1,10 driven *MIH1* gene. Two of each transformant were patched to glucose plates selecting for both copies of chromosome VII and the plasmid. After 1 d of growth, these were replicated to similar selective plates containing sucrose instead of glucose. After another day of growth, these plates were replica plated to complete synthetic media with sucrose (left) or sucrose and galactose (right).
(0.58 MB TIF)

Figure S5 **The interaction between Cdc5 and Rad53.** (A) Western blot of HA-Cdc5 from input and immunoprecipitated Rad53. Strains listed as−/+damage are *CDC13* and *cdc13-1*, respectively. Asterisk denotes *rad53*Δ. (B) In vitro kinase assay performed with purified HA-Cdc5 or kinase dead HA-cdc5-K110A from undamaged or zeocin-treated cells. The substrates (all kinase-dead, D339A) were purified recombinant Rad53 or rad53 R70A R605A (FHA double mutant).
(0.27 MB TIF)

## Abbreviations

APC: Anaphase Promoting Complex
CDK: Cyclin-Dependent Kinase
DSB: double-strand DNA break
FHA: fork-head associated
GFP: green fluorescent protein
IP: immunoprecipitation
ISA: in situ autophosphorylation assay
ssDNA: single-stranded DNA
WT: wild-type
