# Supplementary figures and images for: CDC5 Inhibits the Hyperphosphorylation of the Checkpoint Kinase Rad53, Leading to Checkpoint Adaptation
Source: PLoS Biol. 2010 Jan 26;8(1):e1000286. doi: 10.1371/journal.pbio.1000286 (PMC2811153; doi:10.1371/journal.pbio.1000286)

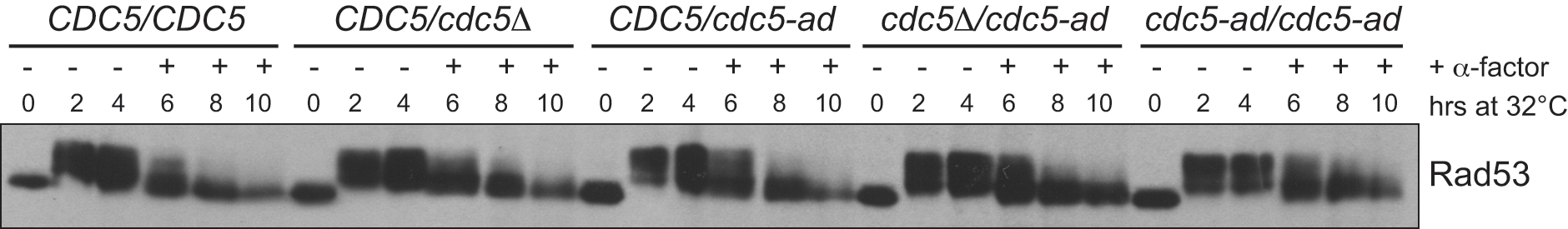

Supplement: Figure S1 — Rad53 phosphorylation in diploids. cdc13-1 strains were grown overnight at permissive temperature (23°C) in rich media containing 2% dextrose, diluted to an OD660 of 0.2, and shifted to 32°C to induce damage. Cells were collected every 2 h. Alpha factor (10 µg/ml) was added to the culture at 4 h with additional boluses at 6 and 8 h to arrest adapting cells in the subsequent G1 phase. Lysates were prepared for Western blot analysis to compare levels of phosphorylated Rad53. yDPT27-2 is CDC5/CDC5; yDPT18-9 is CDC5/cdc5Δ; yDPT28-3 is CDC5/cdc5-ad; yDPT19-19 is cdc5-ad/cdc5Δ; yDPT29-1 is cdc5-ad/cdc5-ad. (0.40 MB TIF) [file pbio.1000286.s001.tif]

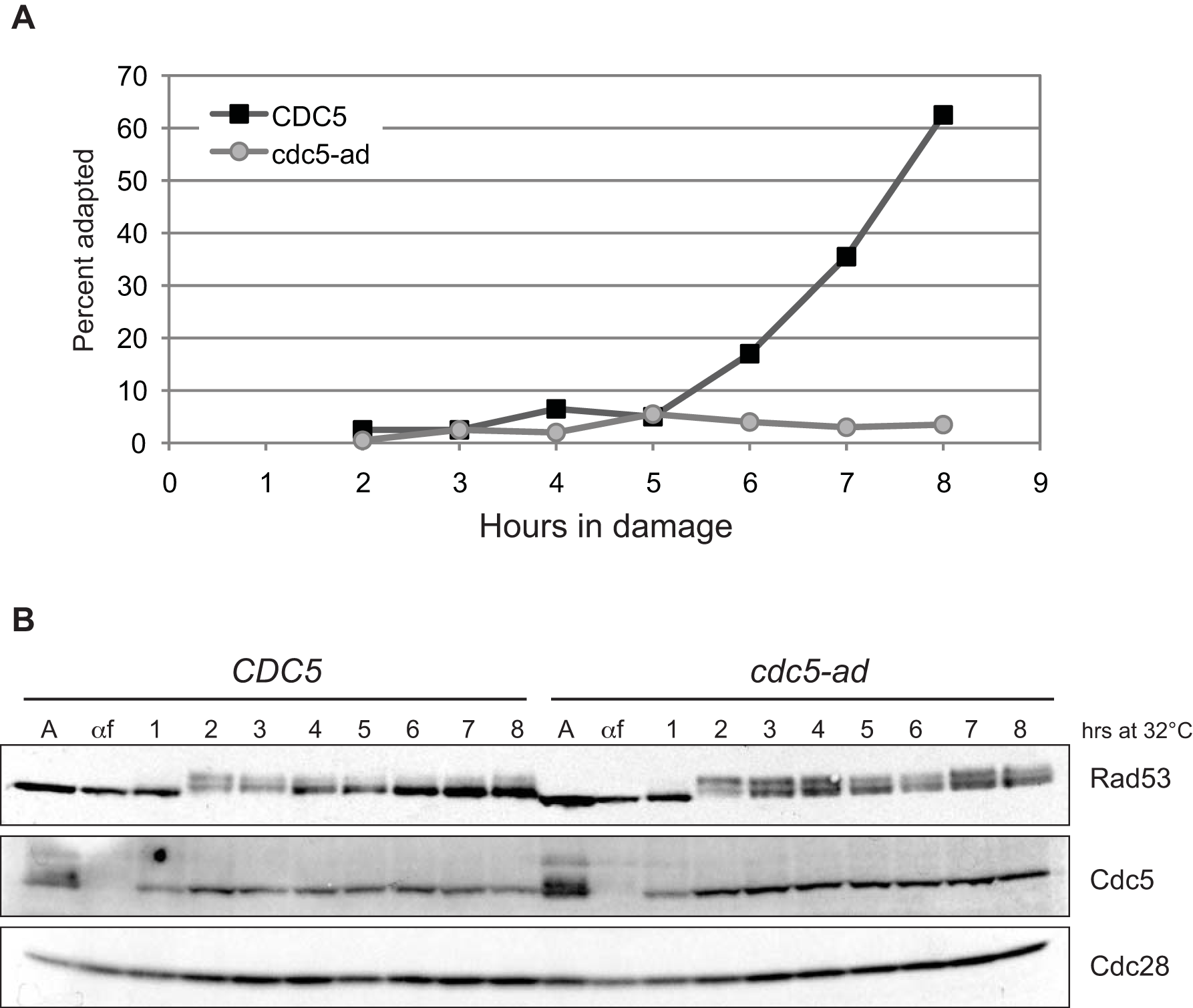

Supplement: Figure S2 — Levels of Cdc5 are unaffected by adaptation. (A) Adaptation was measured by microcolony assay in cdc13-1 CDC5 and cdc13-1 cdc5-ad haploid strains. Cells were initially synchronized in G1 with 7.5 µg/ml of alpha-factor at 23°C for 2 h before release into pre-warmed liquid YM-1 at 32°C to induce damage. Cells were plated 2 h after the temperature shift and counted every hour thereafter. (B) Hourly samples were taken from the adaptation time course described in panel A to measure levels of Rad53, Cdc5, and Cdc28, as a loading control, by Western blot. Asynchronous cells are labeled as A; alpha-factor arrested cells are labeled as αf. (0.47 MB TIF) [file pbio.1000286.s002.tif]

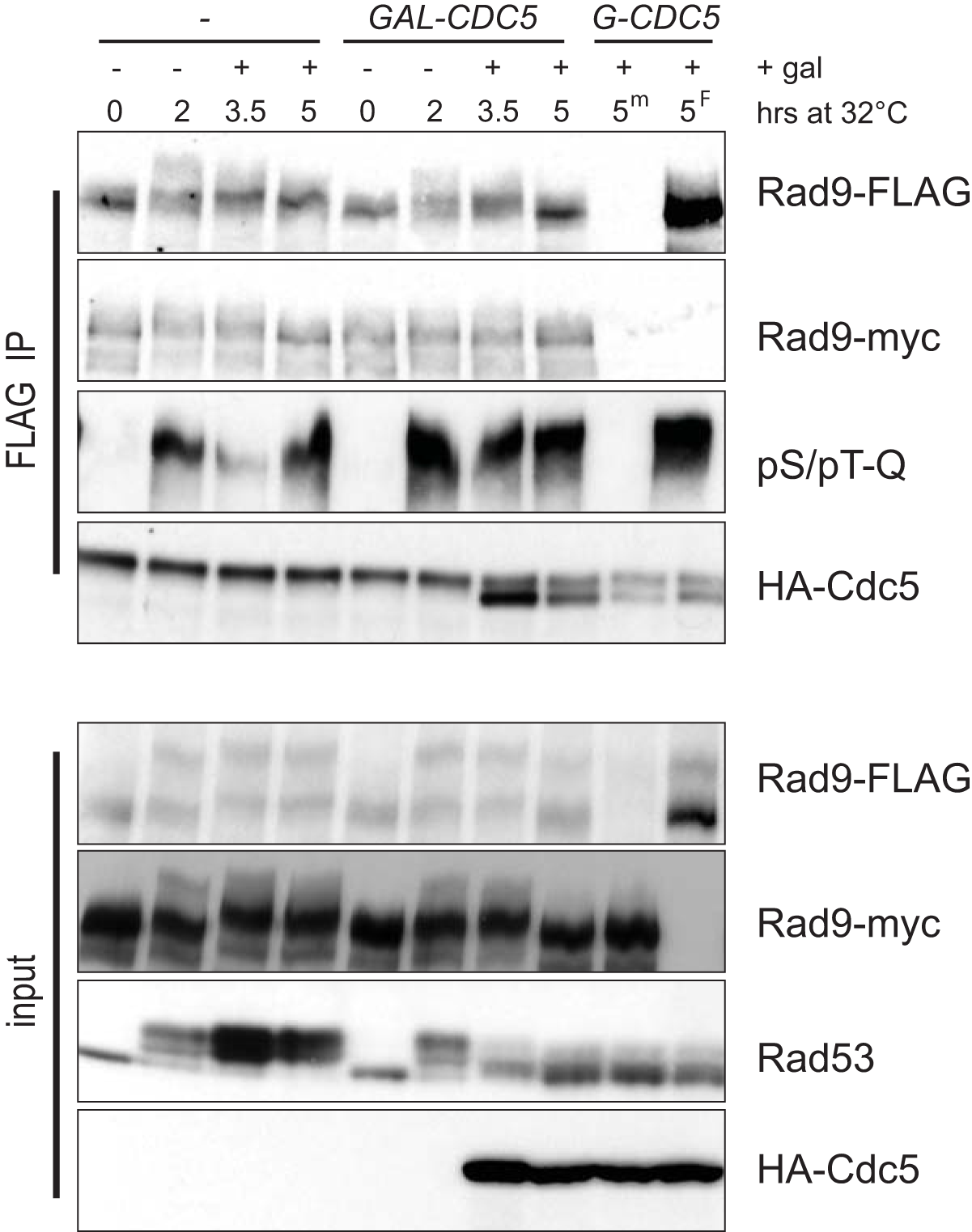

Supplement: Figure S3 — Rad9-Rad9 interaction unaffected by CDC5 overexpression. Rad9-FLAG was immunoprecipitated from strains containing a copy of each RAD9-FLAG and RAD9-18myc that were damaged for 2 h at the non-permissive temperature for cdc13-1, then treated with galactose to induce HA-CDC5. The 5m and 5F denote the 5 h time point of strains that express only RAD9-18myc or RAD9-FLAG, respectively. Input and IP samples were analyzed by Western blotting with the indicated antibodies. (0.58 MB TIF) [file pbio.1000286.s003.tif]

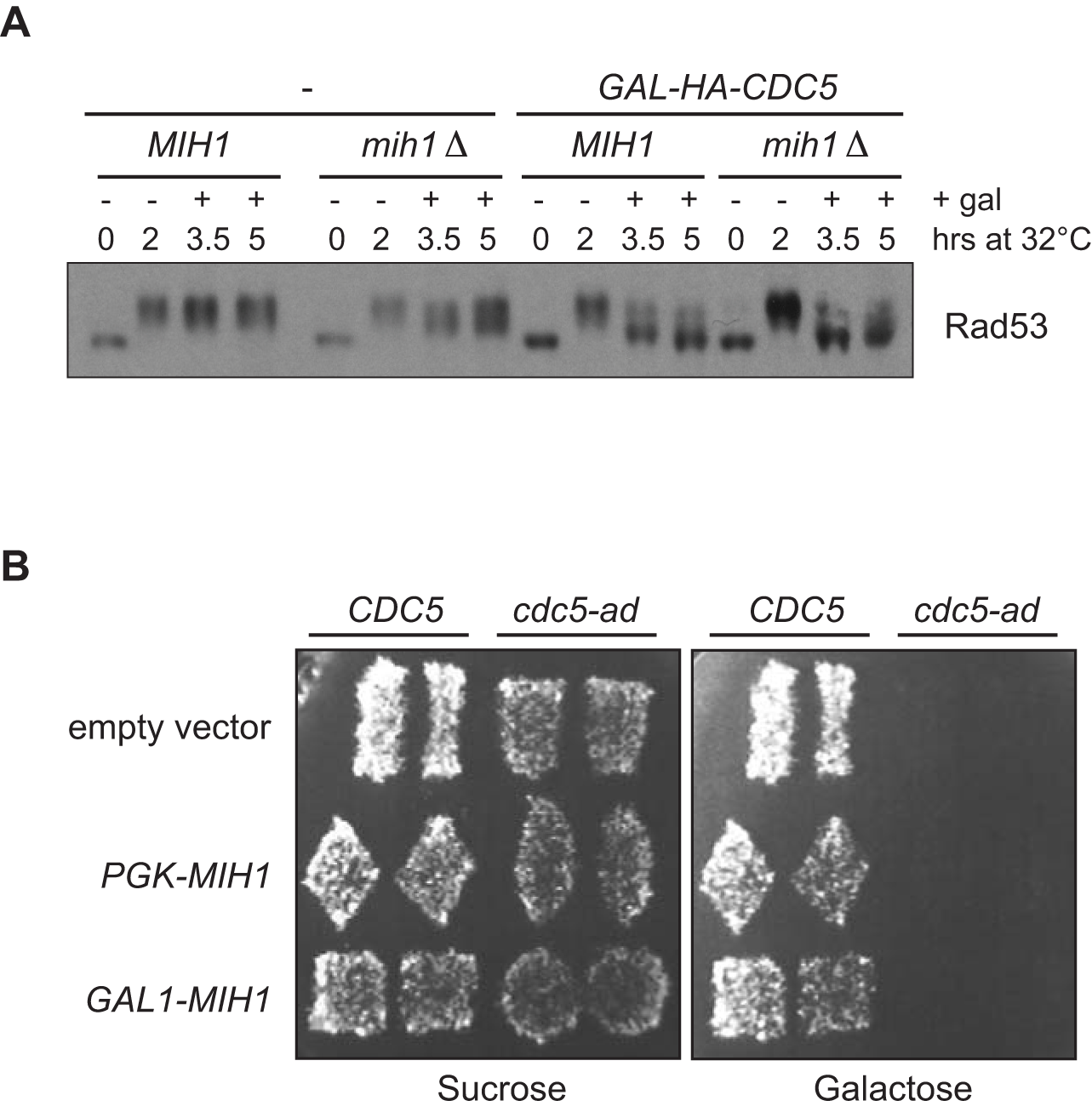

Supplement: Figure S4 — Cdc5 does not regulate adaptation through Mih1. (A) Rad53 phosphorylation was examined after Cdc5 overexpression in wild-type cells, or cells deleted for MIH1, as in Figure 2C. (B) All strains are disomic rad52Δ mutants carrying a galactose-inducible HO endonuclease and a site for the HO endonuclease on the end of a second copy of chromosome VII (see [26],[33] for complete description). CDC5 and cdc5-ad strains were transformed with CEN-based plasmids lacking an insert (“empty vector”) or with a PGK or Gal1,10 driven MIH1 gene. Two of each transformant were patched to glucose plates selecting for both copies of chromosome VII and the plasmid. After 1 d of growth, these were replicated to similar selective plates containing sucrose instead of glucose. After another day of growth, these plates were replica plated to complete synthetic media with sucrose (left) or sucrose and galactose (right). (0.58 MB TIF) [file pbio.1000286.s004.tif]

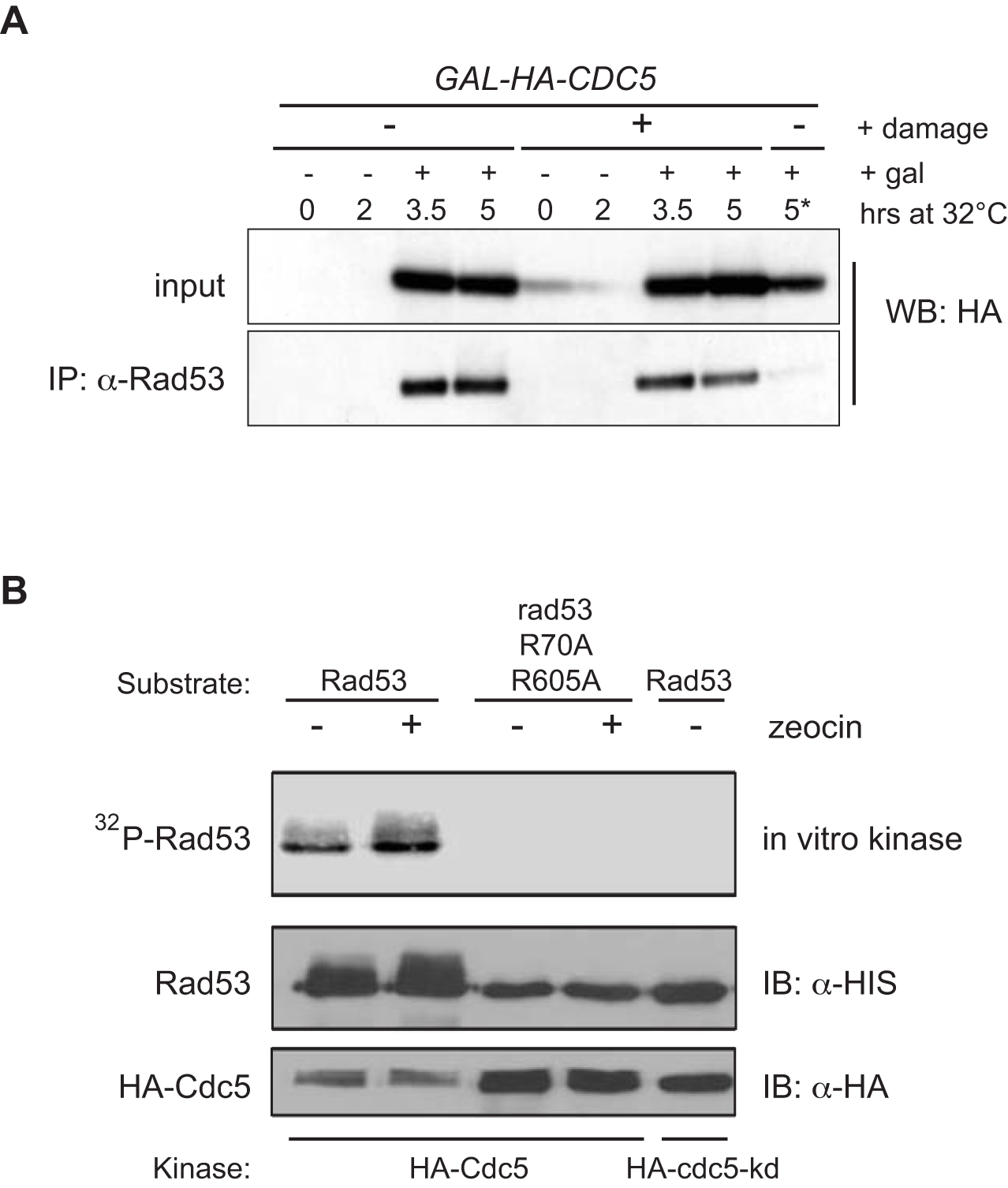

Supplement: Figure S5 — The interaction between Cdc5 and Rad53. (A) Western blot of HA-Cdc5 from input and immunoprecipitated Rad53. Strains listed as−/+damage are CDC13 and cdc13-1, respectively. Asterisk denotes rad53Δ. (B) In vitro kinase assay performed with purified HA-Cdc5 or kinase dead HA-cdc5-K110A from undamaged or zeocin-treated cells. The substrates (all kinase-dead, D339A) were purified recombinant Rad53 or rad53 R70A R605A (FHA double mutant). (0.27 MB TIF) [file pbio.1000286.s005.tif]
